# High-Resolution Computed Tomography of the Temporal Bone in Chronic Otitis Media: An Observational Study at a Tertiary Care Center in Jharkhand, India

**DOI:** 10.7759/cureus.42813

**Published:** 2023-08-01

**Authors:** Abha Kumari, Noman Alam, Sandeep Kumar

**Affiliations:** 1 Department of Pharmacology and Therapeutics, Rajendra Institute of Medical Sciences, Ranchi, IND; 2 Department of ENT, Rajendra Institute of Medical Sciences, Ranchi, IND

**Keywords:** semicircular canal, temporal bone, hrct, ossicular erosion, chronic otitis media

## Abstract

Introduction: Chronic otitis media refers to middle ear inflammation. A radiological exam is a crucial step in this diagnostic process, in addition to a clinical evaluation and an evaluation by an audiologist. For the development of innovative surgical treatment plans for middle ear otitis media with the aim of minimally invasive surgery, accurate information regarding the extent of lesions is required. This is made possible by a temporal bone imaging test.

Materials and methods: The study was conducted at the Rajendra Institute of Medical Sciences (RIMS), a tertiary care facility in Ranchi, Jharkhand, India. The research was conducted between June 1, 2021, and October 31, 2022. In this prospective observational study, 50 patients who visited the otorhinolaryngology clinic at RIMS Ranchi were the participants.

Results: The median age was 26 years, there was a male-to-female ratio of 1.63 to 1, and the vast majority (84%) were from lower socioeconomic classes. High-resolution computed tomography has been shown to be highly sensitive and specific in identifying conditions including scutum erosion, malleus erosion, pneumatization type, mastoiditis, mastoid abscess, and morphological abnormalities such as low-lying dura.

Conclusion: The semicircular canal, fallopian canal, dural plate, and sigmoid sinus may all be clearly defined by non-contrast computed tomography of the temporal bone in erosion in cases of otitis media.

## Introduction

Acute otitis media (AOM), otitis media with effusion (OME; "glue ear"), and chronic otitis media (COM) are three different types of otitis media (OM), or middle ear inflammation. OM may be caused by bacteria or viruses; during "colds," viruses can pass through the eustachian tube to the middle ear and open a pathway for bacteria that live in the nasopharynx [1]. COM is a major contributor to hearing loss in developing nations. Particularly in low-income nations, these complications are dangerous and 21,000 people are thought to pass away from OM complications each year. The prevalence of hearing loss linked to OM is estimated to be 30 (with a range of 0.7-95) per 10,000 people worldwide [1]. The middle ear is housed in the temporal bone, which is surrounded by the meninges, brain, internal carotid artery, jugular bulb, and facial nerve. The anatomical intricacy increases the possibility of COM-related problems, which already makes the surgical process more challenging [2].

Early recognition of COM is crucial to preventing hearing loss and developmental delays or learning difficulties. Radiological examination plays an essential role in the diagnosis of COM, complementing clinical and audiological evaluations. Traditional X-rays have limited utility in evaluating the temporal bone, but computed tomography (CT) scans have become the gold standard imaging modality for the ear, nose, and throat (ENT). CT scans provide detailed information about the bony architecture of the skull base and can also assess soft tissue pathologies associated with bone. They offer excellent resolution of anatomical landmarks, which helps in surgical planning by determining the approach to be used and predicting potential intraoperative complications. The shift toward a minimally invasive surgical approach further highlights the importance of accurate radiological imaging in guiding surgical decisions.

## Materials and methods

The tertiary care hospital Rajendra Institute of Medical Sciences (RIMS) in Ranchi, Jharkhand, India, was the site of the research. Between June 1, 2021, and October 31, 2022, the evaluation was conducted. Fifty patients who saw the ENT doctor at Edges Ranchi were to be included in an impending observational evaluation.

In order to participate in the review, patients suspected of diagnosis of COM were included. Patients who had a previous history of temporal bone neoplasia, ear surgery, or trauma to the head or skull base were not allowed to participate in the research. At RIMS, a high-speed dual-slice CT scanner was used to perform high-resolution computed tomography (HRCT) scans on all of the patients who fulfilled the requirements for inclusion in this study. In response to the findings, actions were taken by both the central and the outer spheres. Scout films of each subject were taken in advance of the actual scan so that any necessary adjustments could be made. Through the use of high-resolution CT, serial sections measuring 1 millimeter in thickness were acquired in both the axial and coronal planes. Major areas of focus included the external tympanic cavity, the tympanic wall, the middle ear, the internal structures of the ear, the path of the facial nerve, the jugular fossa, the sigmoid sinus, the air cells in the mastoid, any bone erosion, and any deficiencies in the semicircular canal. Findings such as erosion of the lateral semicircular canal or erosions of the facial nerve canal are examples of complications that can be attributed to COM.

The evaluation was given the stamp of approval by the Edges Ranchi institution's ethical panel, as noted in the June 25, 2021, memo number 290. We evaluated each patient and correlated our findings with their HRCT scans. We used statistical tools such as sensitivity and specificity to perform an analysis of the statistical significance of the data that were obtained during the course of the study.

## Results

The study included a total of 50 patients according to the inclusion criteria with COM who received treatment at the ENT department of RIMS Ranchi. Here's how the patients' ages broke down: Patients' ages ranged from 11 to 20 years old (42%), 31 to 40 years old (22%), 21 to 30 years old (16%), 51 to 60 years old (8%), 0 to 10 years old (6%), and 41 to 50 years old (6%) were equally represented. Patients' average age was 26.4 years in this research. There were somewhat more male patients than female patients (62% vs. 38%). On the Kuppuswamy social class scale, 16% of the patients were classified as lower middle class, 26% as higher lower class, and 58% as lower class (Table 1).

**Table 1:** HRCT findings for scutum and ossicular erosion HRCT: High-resolution computed tomography

Ossicles /scutum	HRCT	Intraoperative findings	Sensitivity	Specificity
Scutum	41	41	100	100
Malleus	20	20	100	100
Incus	23	26	79.31	100
Stapes	07	04	100	70

Forty percent of the malleus, 46 percent of the incus, and 14 percent of the stapes were degraded in HRCT, and the same percentages were detected intraoperatively. So, the results of the HRCT do not match those obtained after the operation (Table 2).

**Table 2:** HRCT findings for types of mastoid pneumatization HRCT: High-resolution computed tomography; IO: intraoperative

Mastoid	HRCT	IO	Sensitivity	Specificity
Well pneumatized	05	05	100	100
Sclerotic	39	39	100	100
Diploic	06	06	100	100

**Figure 1:**
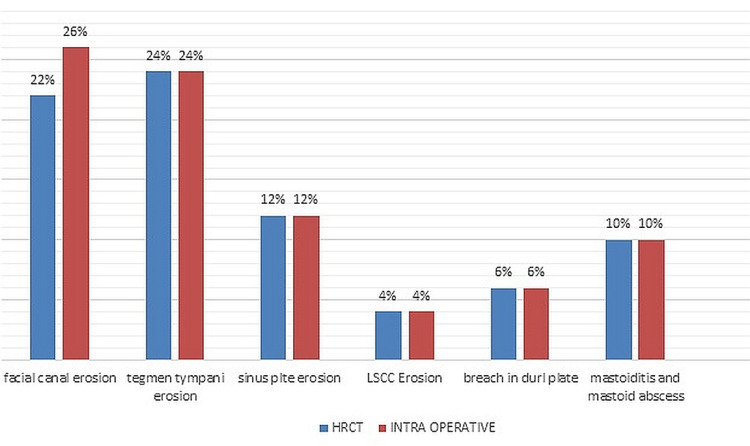
Graph showing complications of COM COM: Chronic otitis media; HRCT: high-resolution computed tomography; LSCC: lateral semicircular canal

Both HRCT and intraoperative examinations revealed that 10% of mastoids were well pneumatized, 78% were sclerotic, and 12% were diploic. Therefore, HRCT has 100% sensitivity and specificity for identifying the kind of pneumatization in the mastoid where sensitivity = [true positive/(true positive + false negative)]x100 and specificity = [True negative/(true negative+ false positive)]x100. HRCT detected facial canal erosion in 22% of patients, whereas intraoperative examination detected it in 26% of patients. Sensitivity was 86.6%, specificity was 100%, and there were just two false negatives. HRCT revealed that 24% of patients had erosion of the tegmen tympani. In 12% of patients, HRCT and intraoperative sinus plate dehiscence were seen. Both HRCT and intraoperative examinations revealed lateral semicircular canal dehiscence in 4% of patients. Ten percent of patients had 100% sensitivity and specificity for detecting sequelae such as mastoiditis and mastoid abscesses using HRCT (Figure 1).

The HRCT findings for the percentage of the middle ear and mastoid structures involved in COM are as follows: 78% epitympanum, aditus, antrum, mastoid air cells, 42% mesotympanum, 40% posterior tympanum, 18% pro tympanum, and 16% hypotympanum (Table 3).

**Table 3:** Sites and extent of involvement of the middle ear and the mastoid air cell system HRCT: High-resolution computed tomography

Extent	HRCT	Intraoperative
Protympanum	09	08
Meso tympanum	21	19
Posterior tympanum	20	19
Epitympanum	39	39
Hypotympanum	08	06
Aditus	37	36
Antrum	36	36
Mastoid air cell	35	35

## Discussion

Fifty patients who were seen at the ENT clinic were selected for the research. Twenty-one (42% of the total) patients fell within the 11-20 age range. The mean age was about 26 years old, which is in line with the findings of Gerami et al. [3], who mentioned a mean age of 27.9 years, and Paperella and Kim [4], who found that the average age is 35 years and one month. The disparity arises because chronic otitis media is more common among American children because of the prevalence of upper respiratory tract infections [5].

In this study, the majority of the patients were males (62%% followed by females (38%), which is in line with Vlastarakos et al. [6] and Gerami et al. [3], where the males affected 71.3% and females 28.8%. The reason for a higher number of males may be that males do most of the outdoor work and are hence more prone to atmospheric and climate change.

Five socioeconomic classes of the modified Kuppuswamy scale for socioeconomic class based on education, occupation of the head of the family, and total per capita income of the family are used in this study. The fact that 92% of the patients in this research were from low-income backgrounds suggests that a lack of cleanliness, a poor diet, and a lowered immune system could be to blame for the disproportionate prevalence of this illness among the poor. Scutum erosion was seen in 82% of individuals with chronic otitis media, the research found. The findings of Rocher et al. are supported by this [7]. In 40% of patients, HRCT successfully identified malleus erosion, which was also observed intraoperatively. In studies by Rocher et al. [7] and Zhang et al. [8], this has been confirmed. Because of its secure affixation to the tympanic membrane, which serves as a mechanical barrier and allows for sufficient blood flow, the malleus experiences far less erosion [9]. HRCT identified incisional erosion in 46% of patients, and intraoperatively it was observed in 52% of patients, which is in agreement with findings by Zhang et al. [8] and Chee et al. [10]. A high percentage of incus erosion is caused by poor blood flow, exposure to the external milieu, especially in posterior perforation, or pressure from severely retracted ear drums [9]. Stapes erosion was observed in 14% of HRCT and 8% intraoperatively, which is comparable to a study by Chee et al. [10].

In HRCT as well as intraoperatively, it was discovered that the mastoid was well-pneumatized in 18%, sclerotic in 78%, and diploic in 4% of cases. Because of this, mastoid pneumatization can be identified by HRCT with 100% accuracy and specificity. This is consistent with a study by Vlastarakos et al. [6], who discovered a substantial correlation between HRCT results and those obtained during surgery for a mastoid-air cell complex.

HRCT detected facial canal dehiscence in 22% of patients with just two false negatives, yielding a sensitivity of 84.61% and a specificity of 100%. Alzoubi et al. [11] and Garber et al. [12] discovered similar results. Tegmen tympani erosion was found in 24% of patients with a sensitivity and specificity of 100%, which is in considerable agreement with Jackler et al. [13]. It is due to lytic enzymes such as collagenase and inflammatory mediators such as TNF alpha, IL-1,1L-6, IL-7, IFN beta, epidermal growth factor TGF-alpha, and matrix metalloproteinase, released by cholesteatoma matrix and granulation tissue [14]. Sinus plate erosion was noted in 12% of patients in HRCT as well as intraoperatively with 100% sensitivity, which is similar to a study by Vlastarakos et al. [6].

Lateral semicircular canal dehiscence was found in 4% of cases, with a sensitivity and specificity of 100%. This is comparable to a study by O'Reilly et al. [15] and Mafee et al. [16], where it was 100% sensitive. The lateral semicircular canal is susceptible to erosion because of its presence in aditus and because it lies in the path of enlarging cholesteatoma [17]. Epitympanum, aditus, antrum mastoid air cells, mesotympanum, posterior tympanum, posterior tympanum, and hypotympanum are all affected to varying degrees by cholesteatoma in HRCT. These percentages are 78%, 72%, 74%, 70%, 42%, 40%, 18%, and 16%, which is in line with the study done by Sirigiri et al. [18].

The study has some limitations, including the need for multicentric studies and additional longitudinal research with a larger sample size.

## Conclusions

For persistent middle ear infections, plain radiography has minimal value. It may help find anomalies in the dural plate, sigmoid sinus plate, and sinodural angle, as well as the kind of mastoid pneumatization. While CT scans are the gold standard, they are not always readily accessible; hence, conventional radiographs are often used in their stead. The ability to clearly see and define anatomical components makes an HRCT scan of the temporal bone an invaluable imaging modality. HRCT serves as a valuable tool, but the expertise and clinical judgment of the surgeons remain essential in achieving optimal outcomes for patients with COM.
